# Fiber mixture-specific effect on distal colonic fermentation and metabolic health in lean but not in prediabetic men

**DOI:** 10.1080/19490976.2021.2009297

**Published:** 2021-12-20

**Authors:** Emanuel E. Canfora, Gerben D.A. Hermes, Mattea Müller, Jacco Bastings, Elaine E. Vaughan, Marco A. van Den Berg, Jens J. Holst, Koen Venema, Erwin G. Zoetendal, Ellen E. Blaak

**Affiliations:** a Human Biology, School for Nutrition and Translational Research in Metabolism (NUTRIM), Maastricht University Medical Center+, Maastricht, The Netherlands; b Laboratory of Microbiology, Wageningen University&Research, Wageningen, The Netherlands; c Sensus BV (Royal Cosun), Roosendaal, Netherlands; d DSM Biotechnology Center, Delft, Netherlands; e NovoNordisk Center for Basic Metabolic Research and Department of Biomedical Sciences Faculty of Health and Medical Sciences, University of Copenhagen, Copenhagen, Denmark; f Centre for Healthy Eating & Food Innovation, Maastricht University - Campus Venlo, Venlo, The Netherlands

**Keywords:** Short-chain fatty acids, dietary fibers, gut microbiota, substrate metabolism

## Abstract

Infusions of the short-chain fatty acid (SCFA) acetate in the distal colon improved metabolic parameters in men. Here, we hypothesized that combining rapidly and slowly fermentable fibers will enhance distal colonic acetate production and improve metabolic health. In vitro cultivation studies in a validated model of the colon were used to identify fiber mixtures that yielded high distal colonic acetate production. Subsequently, in two randomized crossover studies, lean and prediabetic overweight/obese men were included. In one study, participants received supplements of either long-chain inulin+resistant starch (INU+RS), INU or maltodextrin (PLA) the day prior to a clinical investigation day (CID). The second trial studied beta glucan+RS (BG+RS) versus BG and PLA. During each CID, breath hydrogen, indirect calorimetry, plasma metabolites/hormones were assessed during fasting and postprandial conditions. Additionally, fecal microbiota composition and SCFA were determined. In prediabetic men, INU+RS increased plasma acetate compared to INU or PLA (*P* < .05), but did not affect metabolic parameters. In lean men, INU+RS increased breath hydrogen and fasting plasma butyrate, which was accompanied by increased energy expenditure, carbohydrate oxidation and PYY and decreased postprandial glucose concentrations (all *P* < .05) compared to PLA. BG+RS increased plasma butyrate compared to PLA (*P* < .05) in prediabetic individuals, but did not affect other fermentation/metabolic markers in both phenotypes. Fiber-induced shifts in fecal microbiota were individual-specific and more pronounced with INU+RS versus BG+RS. Administration of INU+RS (not BG+RS) the day prior to investigation improved metabolic parameters in lean but not in prediabetic individuals, demonstrating that effects were phenotype- and fiber-specific. Further research should study whether longer-term supplementation periods are required to elicit beneficial metabolic health in prediabetic individuals. Trial registration numbers: Clinical trial No. NCT03711383 (Inulin study) and Clinical trial No. NCT03714646 (Beta glucan study).

## Introduction

Growing evidence indicates that the gut microbiota and its fermentation products may play a crucial role in the etiology of obesity, insulin resistance and obesity-associated type 2 diabetes (T2DM).^1,2-3^ In addition, dietary fibers are increasingly associated with human health.^4^ One of the important activities that the gut microbiota conducts is the fermentation of indigestible carbohydrates such as dietary fibers to the short-chain fatty acids (SCFA) acetate, butyrate, and propionate.^5,6^ In mammals, acetate is produced by almost all members of the gut microbiota and is the most abundant SCFA in the distal gut and systemic circulation.^5^ Several animal studies have shown that an increased acetate availability prevents diet-induced body weight gain, counteracts adiposity, and improves glucose homeostasis and insulin sensitivity in diet-induced obesity.^7–15^ Therefore, it has been suggested that acetate might be a key signaling metabolite in the crosstalk between the gut microbiota and host metabolism. Of note, the SCFA/acetate receptors, G-protein coupled receptor (GPR) 41 and 43, are expressed in nearly all metabolically active tissues of the human body.^5,16^

Evidence from acute human intervention studies supports the potential beneficial effect of acetate administration on substrate and energy metabolism. Acetate administered in the distal colon of normoglycaemic, overweight men increased fat oxidation and circulating peptide YY (PYY). In contrast, no effect on metabolic parameters was seen when acetate was administered in the proximal colon.^17^ In addition, another acute study demonstrated that distal colonic infusions of SCFA mixtures high in acetate increased fat oxidation, energy expenditure and circulating PYY and attenuated whole-body lipolysis in normoglycemic, overweight men.^18^ Thus, the administration/production site of acetate in the colon seems to be of major importance to elicit beneficial metabolic effects.

A straightforward approach to increase acetate availability in the distal colon is the use of acetogenic dietary fibers. However, fermentable fibers with a low molecular mass (e.g. oligosaccharides) are already rapidly fermented in the proximal colon.^19^ Thus, the combination of a ‘rapidly fermentable’ and a complex, more ‘slowly fermentable’ fiber may potentially satiate the proximal microbiota and thereby increase the quantity of the slowly fermentable fiber reaching the distal colon, as has been shown in pigs using combination of resistant starch (as more ‘rapidly fermentable’ fiber) with more complex, steadily fermentable fibers.^19^ A complex, steadily fermentable fiber may be a fiber with a high degree of polymerization and side chains, such as long-chain inulin and beta glucan.

Another important aspect to be considered when studying the effect of gut microbial-derived SCFA on host metabolism is the metabolic phenotype of the study participants. A large body of rodent data suggests a causal link between gut microbiota alterations and metabolic disorders.^20^ In humans, the microbiota of people with obesity and insulin resistance seems to be characterized by a lower microbial diversity as compared to the healthy, lean microbiota.^20,21^ Furthermore, the effect of exogenous- and microbially derived SCFA on the substrate and energy metabolism may differ between metabolic phenotypes. Intervention studies with vinegar (with principal compound acetate) and acetogenic indigestible carbohydrates seem to be more effective in improving glucose homeostasis and insulin sensitivity in metabolically ‘healthy’ individuals compared with individuals with impaired glucose homeostasis, insulin resistance, or T2DM.^22–24^ Interestingly, when acetate was infused intravenously (2.5 mmol/min for 60 minutes) the acetate clearance rate was lower and the half-life time was longer in patients with T2DM than in non-diabetic individuals, indicating a disturbed uptake and/or catabolism of acetate, which might be relevant to elicit acetate-induced metabolic effects in peripheral tissues.^22^ In support, acute intravenously administered acetate led to a greater free fatty acid fall and rebound in healthy compared with hyperinsulinaemic individuals, and this rebound in free fatty acids was negatively correlated with insulin sensitivity indices.^25^

Based on the above findings, we hypothesized that using a mixture of a ‘rapidly fermentable’ (that is fermented by the proximal microbiota) with a ‘slowly fermentable’ acetogenic fiber that reaches the distal colon, will increase acetate availability in the distal colon and systemic circulation, consequently leading to its beneficial metabolic effects. Since gut microbial fermentation capacity and acetate metabolism may differ between metabolic phenotypes both lean and overweight/obese prediabetic individuals were included in this study.

At first, a validated, dynamic, computer-controlled in vitro model of the colon (TIM-2) was used as a screening tool to identify the fiber mixtures that would result in high distal colonic acetate production. Based on these results we conducted two randomized, crossover studies with one day supplementation of two different fiber mixtures (long-chain inulin or yeast beta glucan combined with resistant starch) versus the fibers alone or placebo in lean, normoglycemic as well as overweight/obese, prediabetic men. In the morning after one day of supplement intake, fasting, and postprandial circulating acetate/SCFA concentrations (primary outcomes), breath H_2_, substrate oxidation and energy expenditure, plasma metabolites and hormones as well as fecal microbiota composition were assessed.

## Methods

### In vitro screening

To assess the production of distal acetate/SCFA from different fiber mixtures, TIM-2 was used.^26^ Pooled fecal microbiota samples from obese, prediabetic (n = 14, aged 30–65 years, BMI ≥ 30 kg/m^2^ and ≤ 40 kg/m^2^, fasting plasma glucose > 6.1 mmol/L) and from normoglycemic lean (n = 11, aged 30–65 years, BMI ≥ 20 kg/m^2^ and ≤ 24.9 kg/m^2^, fasting plasma glucose > 5.6 mmol/L) donors recruited in the vicinity of Maastricht, the Netherlands were used in this model. Furthermore, the conditions in the proximal region of the colon, the colon transversum and the distal part of colon were simulated by increasing the pH over a 24-h period (pH 5.8–7.0). Thereby, the transit and fermentation of fibers through the colon were simulated within the first 24 hours of the experiment, whereas the last 16 hours simulated these processes at the more distal colonic site. Three fiber products (7.5 g each) were studied: yeast beta glucan (DSM, Delft, Netherlands), Galacto-oligosaccharides (Vivinal GOS powder, FrieslandCampina Domo, Amersfoort, The Netherlands) and long-chain inulin (Frutafit® TEX!, Sensus B.V., Roosendaal, The Netherlands), with and without the addition of 7.5 g resistant starch (RS2 tapioca starch, Avebe, Veendam, The Netherlands). The substrates were added to TIM-2 as a single shot after a 40-hour incubation of the fecal microbiota in the in vitro model. Samples were subsequently taken at inoculation of the model and 1, 2, 4, 6, 8 and 24 hours after addition of the fiber and SCFA were measured using ion exchange chromatography with conductivity detection (Brightlabs, Venlo, The Netherlands).^27^

### In vivo human studies

#### Participants

For both clinical trials, twelve normoglycemic lean healthy men aged 30–65 years and 12 overweight/obese prediabetic men aged between 30 and 65 years were recruited between February 2018 and May 2019 from the general population in the vicinity of Maastricht, the Netherlands. Only men were included because of the hormonal influences that can take place in female volunteers, which might interfere with the results of this acute study. While studying only males, we get a more homogenous group and therefore we can be sure that the found effect is due to the ingestible carbohydrate mixture/acetate production and not to hormonal influences. During an initial screening visit, eligibility criteria were assessed via anthropometry, an oral glucose tolerance test (OGTT) and a general health questionnaire regarding the participants’ medical history. Inclusion criteria for the lean, normoglycemic group were a BMI between 20 kg/m^2^ and 24.9 kg/m^2^, normal fasting glucose (plasma glucose < 6.1 mmol/L) and glucose tolerance (plasma glucose 2 h after a 75 g glucose drink < 7.8 mmol/L). Inclusion criteria for the overweight/obese prediabetic group were a BMI between 25 kg/m^2^ and ≤ 34.9 kg/m^2^ and impaired fasting glucose (plasma glucose ≥ 6.1 mmol/L and ≤ 7.0 mmol/L) and/or impaired glucose tolerance (plasma glucose ≥ 7.8 mmol/L and ≤ 11.0 mmol/L). All participants were Caucasian, with a systolic blood pressure 100–140 mmHg and diastolic blood pressure 60–90 mmHg, and weight stable for at least 3 months (± 2 kg). Exclusion criteria were: diagnosis of diabetes mellitus, gastroenterological diseases or prior abdominal surgery, cardiovascular diseases, liver or kidney malfunction, patients with a life expectancy shorter than 5 years, participants following a hypocaloric diet, or use of antibiotics, pre- or probiotics in the 3 months prior to start of the study or during the study period. Participants did not use β-blockers, lipid and glucose lowering-drugs, anti-oxidants or chronic corticosteroids. The study was approved by the Medical Ethical Committee of Maastricht University Medical Center+, was conducted in accordance with the Declaration of Helsinki (revised version, October 2008, Seoul, South Korea), and monitored by the independent Clinical Trial Center Maastricht. Written informed consent was obtained from all participants. All authors had access to the study data and reviewed and approved the final manuscript.

### Study design

In these randomized, placebo-controlled crossover studies, participants were allocated to intake of supplements in a random order. The order of intervention was blinded for both the investigator and participants. An independent researcher performed permuted block randomization and assigned participants to interventions. Blinding was ensured by the fact that the content and packaging of the intervention products and placebo sachets looked identical. All supplements were ingested one day prior to a clinical investigational day (CID) with at least a 14-day washout period in-between CIDs. Participants were asked to record a three-day food diary on the three days before each CID. The dietary records were analyzed using the Dutch food composition database (National Institute for public health and environment, Ministry of Health and Welfare and Sport, The Hague, The Netherlands).

The day before the CIDs, participants were instructed to refrain from food products rich in dietary fibers by providing them alternative foods low in fibers (e.g. white bread instead of whole-grain bread). At that day, they received either the fiber (mixture) or a placebo (see interventions). In addition, participants were instructed to refrain from intensive physical activity and alcohol consumption two days prior to the CIDs.

### Interventions

In both studies, three isocaloric supplements were given for a one-day intervention before each CID.

The intervention of the first study (Inulin study) was the one-day consumption of either inulin with maltodextrin (INU), inulin with resistant starch (INU+RS), or a maltodextrin placebo (PLA), which were all isocaloric (43.4 kcal/d). The three supplements were: 1. INU: 12 g (3 × 4 g) long-chain inulin (Frutafit TEX!, Sensus B.V., Roosendaal, The Netherlands) in combination with 5.43 g (3 × 1.81 g) maltodextrin (Glucidex IT 12, Roquette Freres, Lestrem, France) to make it isocaloric. 2. INU+RS: 12 g (3 × 4 g) of long-chain inulin in combination with 9.39 g (3 × 3.13 g (80% resistant starch RS2 (3 × 2.5 g)) granular potato starch (Avebe, Veendam, The Netherlands). 3. PLA: 11.43 g (3 × 3.81 g) maltodextrin.

The intervention of the second study (Beta glucan study) was the one-day consumption of either yeast beta glucan with maltodextrin (BG), BG+RS or PLA, which were all isocaloric (137.1 kcal/d). The three supplements were: 1.BG: 35.25 g (3 × 11.75 g (34% yeast beta glucan (4 g), DSM, Delft, Netherlands) with 5.43 g (3 × 1.81 g) maltodextrin to make it isocaloric. 2. BG+RS: 35.25 g (3 × 11.75 g (34% yeast beta glucan (4 g)) in combination with 9.39 g (3 × 3.13 g (80% RS2 (3 × 2.5 g)) granular potato starch. 3. PLA: 11.43 g (3 × 3.81 g) maltodextrin + 13.1 g protein (3 × 4.37 g) and 4.58 g fat (3 × 1.53 g, same type and amounts as in the beta glucan product).

The fiber (mixture) and placebo were provided to the study participants in sachets. All intervention products were packed at the food-grade kitchen of the Metabolic Research Unit Maastricht by an independent, experienced researcher. The long-chain inulin product was dissolved in hot tea/water before ingestions, while all other products were consumed and mixed with yogurt (0.86 MJ). To ensure blinding in the INU study, PLA was dissolved in hot tea/water in the same manner. In the BG study, all products were mixed in yogurt. Participants were instructed to ingest the supplements with their breakfast, lunch, and a provided standardized dinner low in fibers on the day before their CID. The standardized dinner provided 1.7 MJ, consisting of 62E% carbohydrates, 24E% protein, and 14E% fat. Participants were instructed to ingest the last supplement with the standardized dinner approximately 14 hours before the CID on the next day.

### Clinical investigation days

The day after the fiber/placebo intake, the participants came fasted (>12 hours) to the university for the CIDs. After inserting a cannula into the antecubital vein, blood samples were taken and substrate oxidation and energy expenditure were measured during fasting and after a liquid high-fat mixed meal. The mixed meal provided 2.6 MJ, consisting of 61E% fat (35.5E% saturated fat, 18.8E% monounsaturated fat, and 1.7E% polyunsaturated fat), 33E% carbohydrates and 6E% protein. At fasting and postprandially, breath H_2_ was measured in 30 min intervals using a hand-held breath analyzer (Bedfont EC60 Gastrolyzer, Rochester, UK). In addition, participants sampled feces in the morning of the CIDs.

#### Substrate oxidation and energy expenditure

During the CIDs, energy expenditure, fat, and carbohydrate oxidation were measured using an open-circuit ventilated hood system (Omnical, Maastricht University, The Netherlands). VCO_2_ (L/min) and VO_2_ (L/min) were determined during fasting and at 30, 60, 120, 180, and 240 minutes after the high-fat mixed meal. The equations of Weir^28^ and Frayn^29,30^ were used to calculate resting metabolic rate and the total rate of fat and carbohydrate oxidation, respectively. Nitrogen excretion was calculated based on the assumption that protein oxidation represents 15% of total energy expenditure.

#### Biochemical analyses

During the CIDs, blood was collected into appropriate pre-chilled tubes before and 30, 60, 120, 180, and 240 minutes after a high fat mixed meal intake. The samples were centrifuged at 3,000 g, 4 °C for 15 minutes, plasma was aliquoted and directly snap-frozen in liquid nitrogen and stored at – 80 °C until analysis. Blood was collected into EDTA tubes (Sigma, Dorset, UK) for insulin, glucose, FFA. Plasma FFA and glucose were measured with enzymatic assays on an automated spectrophotometer (ABX Pentra 400 autoanalyzer, Horiba ABX, Montpellier, France). The concentrations of insulin were determined with commercially available radioimmunoassay (RIA) kits (Human Insulin specific RIA, Millipore Corporation, MA, USA). For GLP-1 analysis, blood was collected in a 2 mL EDTA tube containing 20 μL of dipeptidyl peptidase-IV inhibitor (Millipore, Darmstadt, Germany). For PYY analysis, blood was collected in a 2 mL aprotinin tube containing 20 μL of dipeptidyl peptidase-IV inhibitor. Plasma samples were assayed for total GLP-1 immunoreactivity using an antiserum, which reacts equally with intact GLP-1 and the primary (N-terminally truncated) metabolite as previously described.^31^ Total PYY was measured as described previously^28^ using a monoclonal antibody MAB8500 (Abnova, clone RPY-B12), which reacts equally well with PYY_1–36_ and PYY_3–36_. Synthetic human PYY_3–36_ (Bachem, cat no. H-8585) was used as standard and ^125^I-labeled PYY (Perkin Elmer, cat no. Nex341) as tracer. For plasma SCFA, blood was collected in a 4 mL Lithium Heparin tube (BD, Plymouth, UK) and analyzed before and 60, 120 and 240 min after meal intake.^32^ Total concentrations of acetate, propionate and butyrate were measured using gas chromatography (GC) coupled to mass spectrometry (MS) after derivatization of the SCFA with 2,4 difluoroanillin, as described previously.^32,33^

#### Fecal sample collection and determination of fecal SCFA concentration and microbiota composition

In the morning of the CID, fecal samples were collected in tubes for SCFA analysis. Feces were transported using ice packs and immediately stored at −80°C upon arrival at the university. Fecal SCFA was measured using ion exchange chromatography with conductivity detection (Brightlabs, Venlo, The Netherlands) as described for the in vitro experiments and normalized to dry weight.

DNA was isolated from 0.25 g feces with repeated bead beating followed by automated isolation and purification using a Maxwel 16 Tissue LEV Total RNA Purification Kit (Promega, Madison, U.S.). The V4 region of the 16S rRNA gene was amplified with a double barcoded primer pair 515 F (5’-GTGCCAGCMGCCGCGGTAA) – 806 R (5’-GGACTACHVGGGTWTCTAAT).^34^ Each sample was amplified in triplicate using Phusion hot start II high fidelity polymerase (Thermo Scientific, Waltham, U.S.) with the following cycling conditions; Reactions were held at 98°C for 30 s with amplification proceeding for 25 cycles at 98°C for 10 s, 50°C for 10 s, 72°C for 10 s, and a final extension of 7 min at 72°C as previously described.^35^ PCR products were checked for correct size on a 1% agarose gel and subsequently combined and purified with magnetic beads using the CleanPCR kit (CleanNA, Alphen aan den Rijn, The Netherlands). Purified PCR products were quantified with Qubit using the dsDNA BR Assay Kit (Invitrogen, California, USA) and a combined sample for sequencing was created by combining equimolar amounts of amplicons (200 ng) from the individual samples. The resulting library was sent to Eurofins Genomics for 2 × 150nt sequencing on an Illumina Novaseq 6000 instrument. Sequence analysis was performed using NG-Tax 2.0 with default settings.^36^ In short: paired-end libraries were demultiplexed and quality filtered using read pairs with perfectly matching barcodes and allowing a 1 nt mismatch with the amplification primer. Amplicon sequence variants (ASV) were picked as follows: sequences were ordered by abundance per sample and reads were considered valid when their cumulative abundance was ≥ 0.1%. Taxonomy was assigned using the SILVA reference database version 128.^37^ ASVs are defined as unique sequences rather than a cluster of sequence variants with a shared similarity above a specified threshold, generally 97%, such as operational taxonomic units. Sequence data have been deposited in the European Nucleotide Archive, accession number PRJEB47404.

### Sample size calculation

Based on our previous study^18^ using a randomized, crossover design and a comparable statistical model for analysis it was estimated that an increase of 30% (with an SD of 5) in circulating acetate concentrations is necessary to detect a physiologically relevant metabolic effect. Using Gpower (Version 3.1 for Mac, Parkville, Victoria, Australia), it was calculated that 9 participants per study group were sufficient to detect this difference in acetate levels between treatments considering a power of 80% at an alpha level of *P* = .05. Considering a putative dropout rate of 20%, 12 lean, and 12 overweight/obese prediabetic individuals were recruited for both studies.

### Statistical analysis

In both human intervention studies, differences between baseline characteristics of lean versus overweight/obese prediabetic participants were analyzed using a Student’s independent-samples t-test. Postprandial responses in energy expenditure, substrate oxidation, metabolites, and hormones were expressed as total area under the curve (AUC0-240) or divided in periods of 2 h (AUC0-120 and AUC 120–240). Differences in fasting (t0) and postprandial AUC between the three interventions (PLA, INU, and INU+RS or PLA, BG, and BG+RS) were analyzed using a linear mixed model for repeated measures. Intervention was set as fixed factor and participants were set as a random factor. Although no carry-over effects were expected due to the 14-day washout period, we tested for carry-over effects by adding “period” (order of treatments) to the model as fixed factor and tested for its significance. In both trials, no carry-over effect was present in the assessed parameters. Post hoc comparison of treatment groups was performed, when the overall treatment effect was at least *P* < .1. Due to the explorative nature of these studies, least significant testing (LSD) was used for the post hoc comparison. All statistics were performed using SPSS 25.0 for Macintosh, *P* < .05 (two-sided *P*-value) was considered statistically significant.

For microbiota composition, all analyses were performed in R version 3.4.0. We calculated beta-diversity using weighted UniFrac and unweighted UniFrac, which are based on the phylogenetic relatedness of the ASVs. While weighted UniFrac takes into account the abundance of each ASV, unweighted UniFrac provides equal weight to all ASVs, thereby focusing on the presence or absence of low abundance ASVs.^38^ Principal coordinate analysis (PCoA) was performed on the resulting matrices to visualize the multivariate effects of the intervention on microbiota using the phyloseq^39^ and ggplot2^40^ packages. Within individuals, UniFrac distances were calculated to determine the effect sizes of both fiber interventions on the microbiota compared to the placebo. A paired students t-test was used to determine the significance between the addition of RS within each fiber intervention, while an unpaired test was used to determine the significance between fiber interventions. We calculated Shannon diversity and ASV richness to define microbial alpha-diversity for each participant as implemented in phyloseq. To determine whether there were pairwise differences in diversity between groups, we performed an ANOVA and Tukey’s Honest Significant Difference post hoc test, corrected for multiple testing. An adjusted *P* < .05 was considered significant. For all other tests, a *P*-value of < 0.05 was considered significant.

## Results

### In vitro screening

In the validated in vitro model TIM-2, the addition of RS to INU resulted in an increased cumulative acetate, butyrate, and total SCFA production in the last 16 h (simulating production in the distal colon) versus INU alone (supplementary figure 1) using microbiota of lean, but not when microbiota of overweight/obese prediabetic individuals was used. In contrast, co-fermentation of RS+GOS did not increase distal colonic acetate production as compared to fermentation with GOS only, neither in lean nor in prediabetic individuals (supplementary figure 2). Moreover, INU as well as INU+RS showed greater acetate and total SCFA production in the simulated distal colon in prediabetic individuals (supplementary figure 1) as compared to GOS and GOS+RS (supplementary figure 2). The production rates and ratios of the three SCFA acetate, propionate, and butyrate differed after fermentation of INU and GOS (with or without the addition of RS) when compared to BG. Whereas the former lead to acetate > butyrate ≫ propionate, the distribution after BG fermentation shifts to acetate > propionate ~ butyrate. In addition, BG combined with RS increased late acetate and total SCFA production in the simulated distal colon using microbiota of lean and prediabetic individuals (supplementary figure 3) compared to BG alone and GOS+RS. Based on these results, which show an increased distal colonic acetate production using INU+RS and BG+RS in lean individuals, we chose to investigate the effect of INU+RS versus INU and PLA as well as BG+RS versus BG and PLA on circulating acetate/SCFA concentrations and substrate and energy metabolism in lean as well as in overweight/obese, prediabetic men in two in vivo crossover studies.

### In vivo studies

#### Inulin study

Twelve normoglycaemic lean and 12 overweight/obese, prediabetic men were included in this intervention study (Table 1). One participant in the prediabetic group did not continue the study after his first CID due to personal circumstances. The lean and prediabetic men were comparable in age, but differed in BMI, fasting glucose concentrations and glucose concentrations two hours after the glucose load.

**Table 1.** Study participants’ baseline characteristics (n = 45)

	Inulin study					Beta glucan study				
	Lean (n = 12)		Prediabetic (n = 11)			Lean (n = 11)		Prediabetic (n = 11)		
Variables	**Mean**	**SD**	**Mean**	**SD**	*P*-value	**Mean**	**SD**	**Mean**	**SD**	*P*-value
**Age, years**	54	12	59	7	0.197	59	8	61	7	0.648
**Height, m**	1.76	0.08	1.79	0.06	0.398	1.77	0.07	1.8	0.07	0.328
**Weight, kg**	73.5	9.3	93.3	10.3	<0.001	76.1	7.8	96.5	12.6	<0.001
**Body Mass Index, kg/m^2^**	23.6	1.4	29.2	2.8	<0.001	24.1	0.9	29.7	3.0	<0.001
**Waist (cm)**	86.8	7.2	107.3	9	<0.001	92.5	4.6	109.8	6.6	<0.001
**Hip (cm)**	96.3	6.3	107.1	7.3	0.001	95	5.64	105.6	5.65	<0.001
**Waist-Hip Ratio**	0.90	0.05	1.00	0.05	<0.001	0.97	0.05	1.04	0.03	0.001
**Systolic blood pressure, mmHg**	125	11	131	13	0.227	124	14	133	9	0.120
**Diastolic blood pressure, mmHg**	79	7	79	8	0.828	80	9	84	5	0.169
**Fasting glucose, mmol/L**	5.12	0.23	5.97	0.75	0.003	4.93	0.33	6.28	0.68	<0.001
**OGTT 2 h glucose, mmol/L**	4.78	1.33	8.1	2.52	0.002	3.92	1.18	8.02	2.24	<0.001
**HbA1c %**	5.3	0.3	5.6	0.3	0.022	5.2	0.2	5.5	0.2	0.002
**HbA1c mmol/mol Hb**	34	3	38	4	0.004	33	2	37	2	0.001

Notes: Values are presented as mean ± standard deviation (SD). Differences between baseline characteristics of lean versus prediabetic participants were analyzed using a Student’s independent-samples t-test.

Abbreviations: BMI, body mass index; OGTT, oral glucose tolerance test; ALAT, alanine-aminotransferase; Hb1Ac, hemoglobin 1Ac.

#### Beta glucan study

Twelve normoglycaemic lean and 12 overweight/obese, prediabetic men were included in this intervention study (Table 1). One participant in the lean and one in prediabetic group did not continue the study after the first CID due to personal circumstances. The lean and prediabetic men were comparable in age but differed in BMI, fasting glucose concentrations and glucose concentrations two hours after the glucose load.

No serious adverse events occurred during both clinical trials. We asked the participants to report whether they experience Gastrointestinal symptoms after the intake of the fiber(mixtures) or placebo. In both trials, none of the volunteers experienced abdominal cramps, rumbling or bloating.

In the INU trial, two lean individuals experienced increased rate of flatulence (one after INU intake one after INU+RS) and two other lean participants increased rate of bloating after INU intake. One prediabetic participant reported flatulence after INU intake.

In the BG trial, one lean participant reported increased rates of flatulence and bloating after BG. Two other participants report flatulence after BG+RS. One prediabetic participant reported increased rates of flatulence after PLA, BG, and BG+RS and another prediabetic participant reported increased flatulence and bloating after BG intake.

Of note, all the above-mentioned participants reported that the increased flatulence and bloating was not present anymore in the morning of the CID.

Compliance for fiber intake for both studies was 100% as checked by empty sachets returned, and food diaries (see supplementary data 1 and supplementary table 1).

### Plasma and fecal SCFA concentrations, and breath H_2_

**Figure 1.**
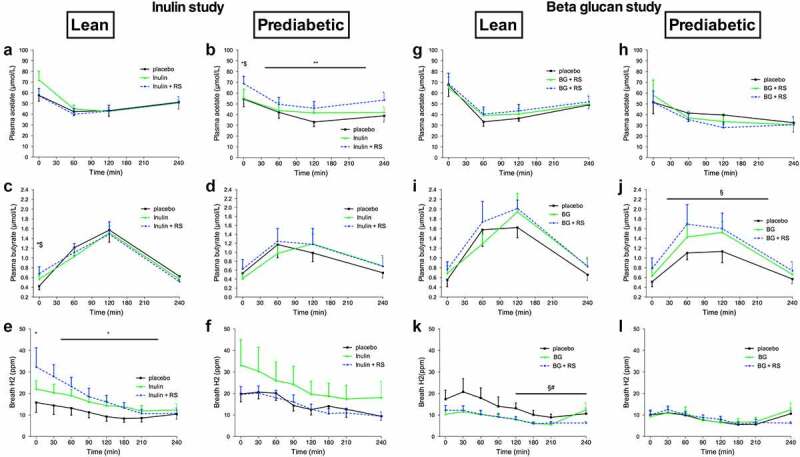
Plasma short-chain fatty acids and breath hydrogen before and after a high fat mixed meal after INU, INU+RS and PLA in lean (n = 12) and prediabetic (n = 11) men as well as BG, BG+RS and PLA supplementation in lean (n = 11) and prediabetic (n = 11) men. Plasma acetate of lean (a) and prediabetic (b), plasma butyrate of lean (c) and prediabetic (d), and breath H_2_ excretion of lean (e) and prediabetic (f) men in the inulin study. Plasma acetate of lean (g) and prediabetic (h), plasma butyrate of lean (i) and prediabetic (j), and breath H_2_ excretion of lean (k) and prediabetic (l) men in the beta glucan study. Values are means ± S.E.M. Differences in fasting (t0) and postprandial AUC between the three interventions (PLA, INU, and INU+RS or PLA, BG and BG+RS) were analyzed using a linear mixed model for repeated measures and the indicated *P*-values represents post-hoc testing

Data on plasma SCFA and breath H_2_ are presented in supplementary table 2 and Figure 1. Data on fecal SCFA are presented in supplementary figure 4.

*P < .05 INU+RS versus placebo, **P < .01 INU+RS versus placebo, $P < .05 versus INU, §P < .05 BG+RS versus placebo, #P < .05 BG+RS versus BG.

BG, beta glucan, RS, resistant starch; INU, long-chain inulin; PLA, placebo.

#### Inulin study

Fasting and postprandial plasma acetate concentrations were not different between treatment groups in lean men (Figure 1a). In prediabetic individuals, one day INU+RS intake increased the next morning fasting plasma acetate concentrations versus PLA (*P* = .033) and INU (*P* = .043) as well as postprandial acetate concentrations compared to PLA (AUC0-240, *P* = .006, Figure 1b).

In lean participants, fasting plasma butyrate concentrations were higher with INU+RS as compared to PLA (*P* = .025) and INU (*P* = .043, Figure 1c). In prediabetic men, fasting and postprandial plasma butyrate concentrations did not change upon intervention (Figure 1d).

Fasting and postprandial plasma propionate did not differ between treatment groups in both phenotypes.

No significant differences in fecal total SCFA, acetate, propionate, and butyrate were detected between treatment conditions neither in lean nor in prediabetic participants.

In the lean group, breath H_2_ excretion during fasting (*P* = .017) and postprandial (AUC0-240, *P* = .016) conditions was higher in the INU+RS as compared to PLA (Figure 1e). In prediabetic participants, no differences between the interventions in both fasting and postprandial breath H_2_ were found (figure 1f).

#### Beta glucan study

In lean and prediabetic men, fasting, and postprandial acetate (Figure 1g and h) and propionate concentrations did not differ between treatment groups.

In prediabetic men, postprandial butyrate concentrations were higher with BG+RS as compared to PLA (AUC0-240, *P* = .013, Figure 1j) and were not different between treatment groups during fasting conditions. Fasting and postprandial plasma butyrate concentrations did not differ between treatment groups in lean men (Figure 1i).

Fasting and postprandial plasma propionate did not differ between treatment groups in both phenotypes.

As for the Inulin study, no differences in fecal total SCFA, acetate, propionate, and butyrate were detected between treatment conditions neither in lean nor in prediabetic participants.

In the lean group, breath H_2_ excretion during the last two hours of postprandial conditions was lower in the BG+RS (AUC120-240, *P* = .012) and BG (AUC120-240, *P* = .010, Figure 1k) group as compared to PLA. In prediabetic participants, no differences between the interventions in both fasting- and postprandial breath H_2_ were found (Figure 1l).

### Energy expenditure and substrate oxidation

**Figure 2.**
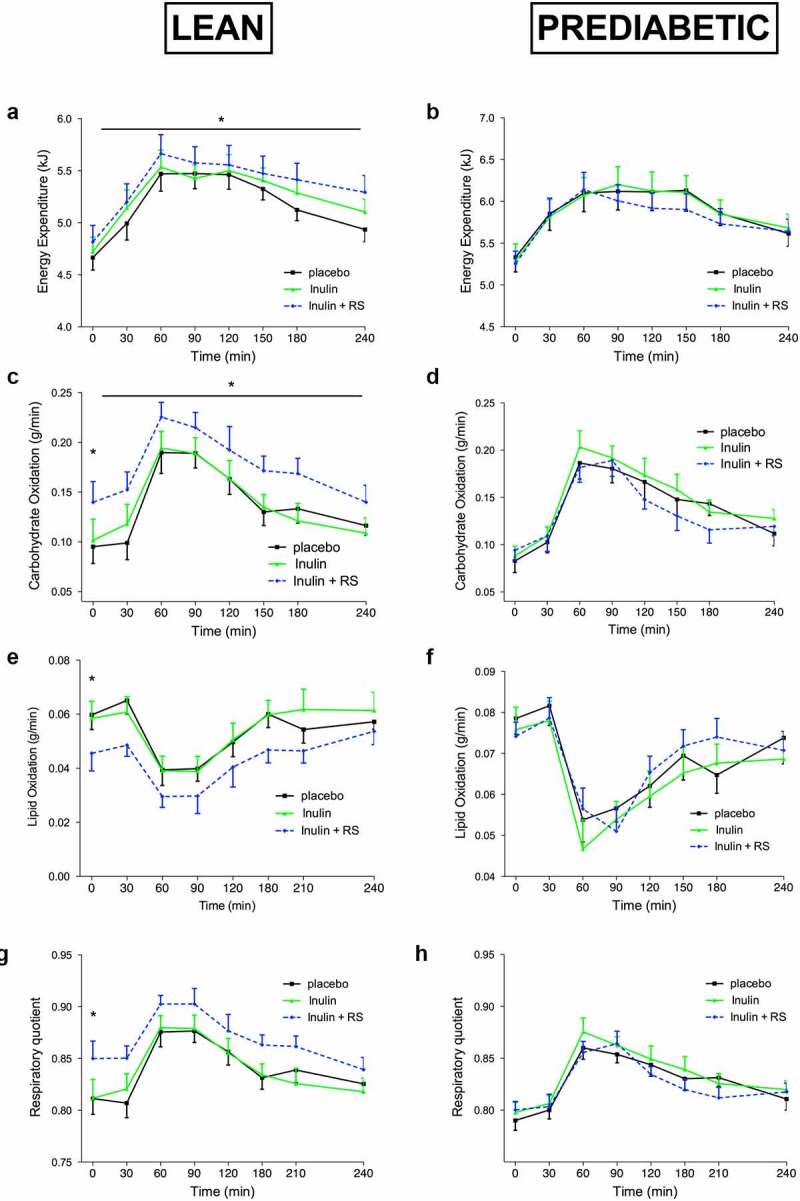
Energy Expenditure and Substrate Oxidation before and after a high fat mixed meal after INU, INU+RS and PLA supplementation in lean (n = 12) and prediabetic (n = 11) men. Energy expenditure of lean (a) and prediabetic (b), carbohydrate oxidation of lean (c) and prediabetic (d), fat oxidization of lean (e) and prediabetic (f) and respiratory quotient of lean (g) and prediabetic (h) men. Values are means ± S.E.M. Differences in fasting (t0) and postprandial AUC between the three interventions (PLA, INU, and INU+RS) were analyzed using a linear mixed model for repeated measures and the indicated *P*-values represents post-hoc testing

Data on energy expenditure and substrate oxidation are presented in supplementary table 3 and Figure 2.

*P < .05 INU+RS versus PLA.

RS, resistant starch; INU, long-chain inulin; PLA, placebo.

#### Inulin study

In lean participants, postprandial energy expenditure (AUC0-240, *P* = .020 Figure 2a), as well as fasting (*P* = .025) and postprandial (AUC0-240, *P* = .042) carbohydrate oxidation (Figure 2c) increased after INU+RS intake compared to PLA. Fasting fat oxidation decreased after INU+RS compared to PLA in lean individuals (*P* = .029, Figure 2e). In line, fasting RQ was higher the morning after INU+RS intake compared to PLA (*P* = .026, Figure 2g).

No differences in resting and postprandial energy expenditure, carbohydrate, and fat oxidation nor in RQ were detected between treatment conditions in the prediabetic participants (Figure 2 B, D, F, and H).

#### Beta glucan study

No treatment effects on fasting and postprandial energy expenditure, carbohydrates and fat oxidation between BG+RS compared to BG alone or PLA were found in lean or prediabetic participants.

### Plasma metabolites and hormone concentrations

**Figure 3.**
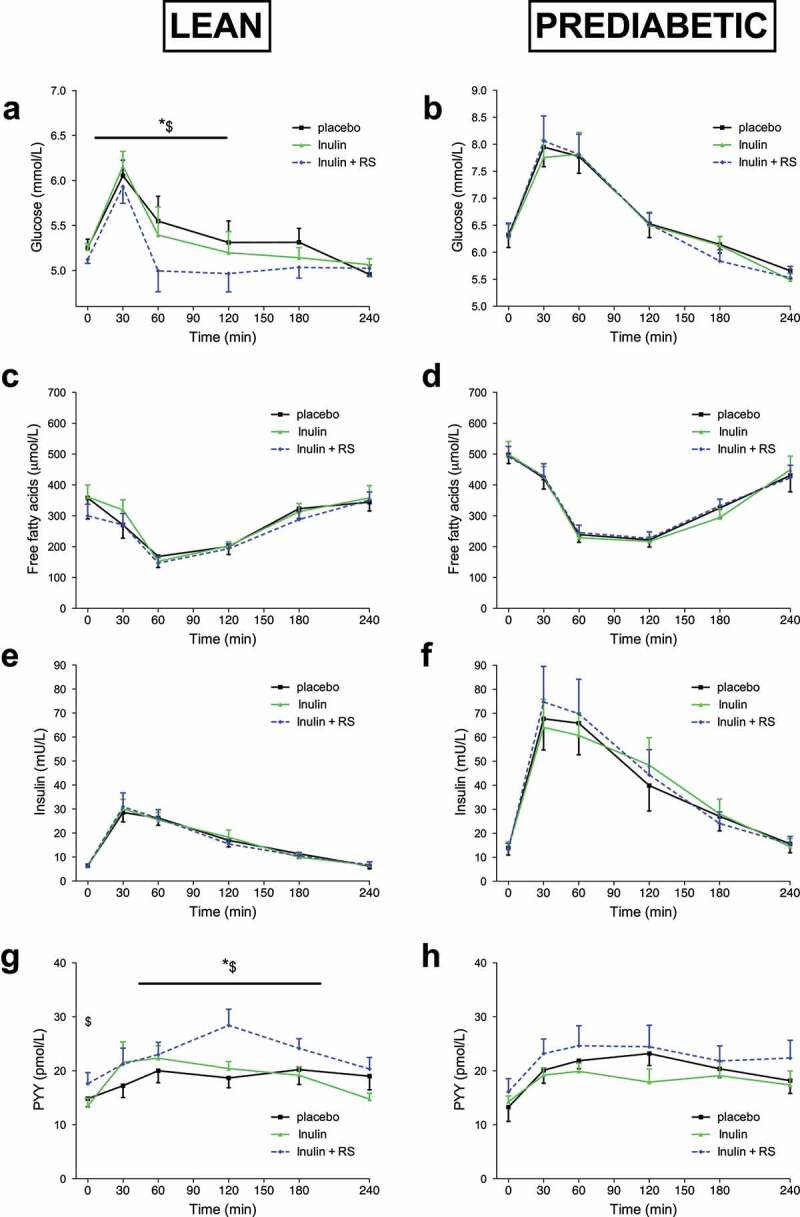
Plasma metabolite and insulin concentrations before and after a high fat mixed meal after INU, INU+RS and PLA supplementation in lean (n = 12) and prediabetic (n = 11). Plasma glucose concentrations of lean (a) and prediabetic (b), FFA concentrations of lean (c) and prediabetic (d), insulin concentrations of lean (e) and prediabetic (f), PYY concentrations of lean (g) and prediabetic (h) individuals. Values are means ± S.E.M. Differences in fasting (t0) and postprandial AUC between the three interventions (PLA, INU, and INU+RS) were analyzed using a linear mixed model for repeated measures and the indicated *P*-values represents post-hoc testing

Data on plasma metabolites and hormone concentrations are presented in Figure 3 and supplementary table 4.

*P < .05 INU+RS versus PLA; $P < .05 INU+RS versus INU.

RS, resistant starch; PYY, peptide YY; INU, long-chain inulin; PLA, placebo.

#### Inulin study

In lean individuals, INU+RS decreased postprandial glucose concentrations in the first 2 hours after ingestion of the meal as compared to INU (AUC0-120, *P* = .028) and PLA (AUC0-120, *P* = .022, Figure 3a). In contrast, no significant treatment effects on plasma glucose levels were found in prediabetic participants (Figure 3b). Plasma FFA did not differ between treatments in both groups (Figure 3c and d). Plasma insulin did not differ between treatments in lean (Figure 3e) nor in prediabetic (figure 3f) participants. The reduced glucose concentrations in the early postprandial phase with INU+RS as compared to INU and PLA with similar insulin concentrations suggests an improved postprandial insulin sensitivity in lean individuals.

One day INU+RS intake increased fasting plasma PPY concentrations on the next morning as compared to INU (*P* = .012) and postprandial PYY concentrations as compared to INU (AUC0-240, *P* = .024) and PLA (AUC0-240, *P* = .016) in lean men (Figure 3g). Plasma PYY concentration did not differ between treatment groups in prediabetic men (Figure 3h).

Plasma GLP-1 did not differ between treatments during fasting and postprandial conditions in both groups.

#### Beta glucan study

Plasma FFA concentrations were lower after BG+RS as compared to BG during the last two hours of postprandial conditions in prediabetic men (AUC120-240, *P* = .027). No further significant treatment effects on plasma metabolites and hormones between BG+RS compared to BG alone or PLA were found in lean nor prediabetic participants.

### Fecal microbiota profile

**Figure 4.**
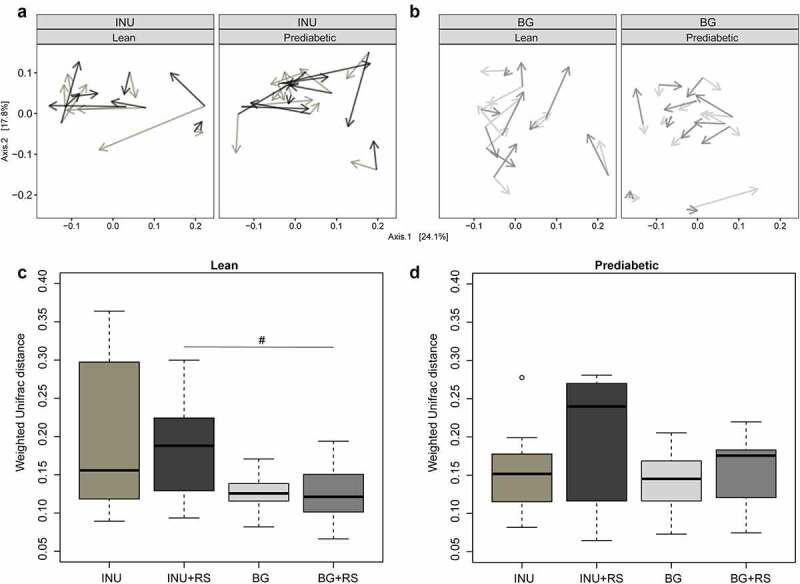
Changes in fecal microbiota composition (weighted and unweighed UniFrac) after INU, INU+RS and PLA in lean (n = 8, four individuals were not able to sample feces on all 3 days) and prediabetic (n = 10, one individual was not able to sample feces on all day) men as well as after BG, BG+RS and PLA supplementation in lean (n = 10, one individual was not able to sample feces on all day) and prediabetic (n = 11) men. A & B: PCoA plots of weighted (a) and unweighted UniFrac (b): the longer the arrow, the larger the change. The start point for each arrow is the composition after PLA intervention while lighter gray depicts the change due to INU, darker gray to INU+RS, lighter gray BG and darker gray BG+RS. The random direction of the arrows after each intervention shows the individual effects. C & D: Intra-individual microbiota changes compared to placebo after fiber intervention in lean (c) and prediabetic (d) men

Sequencing of the V4 region of the 16S rRNA gene resulted in an average (±SD) of 212835(±108736) reads/sample. Weighted UniFrac-based PCoA showed that the direction of the change in microbiota composition was specific for each individual (Figure 4; and for the individual changes in abundance of the top 12 bacterial families/genera supplementary figure 5). The addition of RS to INU tended to generally change the microbiota composition more than INU alone in prediabetic (*P* = .1) but not in lean (*P* = .6) individuals. The change in microbiota composition after the intake of BG and BG+RS was small and did not differ between BG and BG+RS in lean (*P* = 1.0) and prediabetic (*P* = .55) men. Overall, INU+RS tended to change the microbial composition more than BG+RS, which almost reached significance in lean (*P* = .06) but not in prediabetic individuals (*P* = .13).

#P = .06 INU+RS versus BG+RS.

RS, resistant starch; BG, beta glucan; INU, long-chain inulin; PLA, placebo.

**Figure 5.**
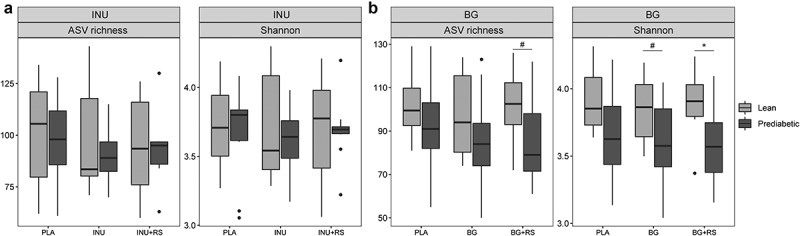
Impact of (a) INU, INU+RS and placebo (b) BG, BG+RS and placebo supplementation on microbiota alpha diversity (ASV richness and Shannon) of lean (light gray) and prediabetic (dark gray) individuals. The fecal microbiota of 8 lean (four individuals were not able to sample feces on all 3 days) and 10 prediabetic (one individual was not able to sample feces on all day) men in the inulin study and 10 lean (one individual was not able to sample feces on all day) and 11 prediabetic men in the beta glucan study was used

For INU microbial alpha-diversity showed large variation between participants and no uniform change. However, within the BG intervention Shannon-diversity of lean men was unaffected, while BG+RS tended to lead to lower Shannon-diversity (*P* = .08) as compared to PLA in prediabetic men (Figure 5).

*P < .05, #P < .1.

RS, resistant starch; BG, beta glucan; INU, long-chain inulin; PLA, placebo.

## Discussion

In these two double-blind, placebo-controlled, randomized, crossover studies, the acute effects of one-day consumption with a ‘slowly fermentable’ complex INU or BG alone, or INU or BG combined with a more ‘rapidly fermentable’ RS on substrate and energy metabolism were studied. Here, fecal and plasma SCFA concentrations, breath H_2_ excretion, as well as fasting and postprandial energy expenditure and substrate oxidation and circulating metabolites/hormones the morning after the supplementation day in lean and prediabetic overweight/obese men were assessed. These fiber mixtures were selected based on a high distal colonic acetate and total SCFA production in a validated in vitro model of the human colon, which was hypothesized to impact human substrate and energy metabolism in vivo. Briefly, in lean men, INU+RS increased fasting plasma butyrate, breath H_2_, energy expenditure and carbohydrate oxidation, improved postprandial insulin sensitivity and increased plasma PYY concentrations when compared to PLA. In contrast, INU+RS increased postprandial circulating acetate concentrations compared to PLA and INU in overweight/obese, prediabetic men, but did not affect breath H_2_, energy expenditure and substrate metabolism. BG+RS increased postprandial plasma butyrate concentrations compared to PLA, in prediabetic, but not lean, men. However, BG+RS did not affect other fermentation markers or metabolic outcomes when compared to BG alone or placebo. Fecal microbiota was affected by both treatments, but the shift in composition was highly individually specific and tended to be more pronounced with INU+RS as compared to BG+RS.

In lean individuals, an increase in microbial fermentation markers (breath H_2_ and fasting plasma butyrate) was coincident with increases in energy expenditure and carbohydrate oxidation, increased circulating PYY concentrations and improved postprandial insulin sensitivity after INU+RS versus PLA. These outcomes may suggest that the fiber-induced metabolic effects are mediated by gut microbial SCFA production. In line with these findings, SCFA have been shown to improve host energy expenditure, insulin sensitivity, and glucose tolerance in several animal studies. For example, supplementation of sodium acetate or butyrate to a high-fat diet reduced body weight gain and improved insulin sensitivity in mice.^14,41^ In addition, knockout of the SCFA receptor GPR43 in high fat fed mice resulted in an abolished anti-obesogenic effect of inulin that was demonstrated in wild-type littermates.^9^ The underlying mechanisms involve SCFA-induced gut-brain signaling pathways including increased secretion of gut hormones as well as direct effects of SCFA on peripheral metabolism in tissues such as the adipose tissue and skeletal muscle.^3^ Furthermore in line with our results in lean individuals, a human study with lean individuals showed that the consumption of a whole grain flour and rye kernels bread, both of which contain inulin, combined with RS in the evening increased breath H_2_ excretion, decreased postprandial glucose and insulin responses and increased circulating PYY concentrations the next morning as compared to a white wheat flour bread.^42^

In contrast to the results in lean individuals, supplementation of INU+RS did not increase breath H_2_ and circulating butyrate concentrations in the prediabetic group. Interestingly, circulating acetate concentrations increased after INU+RS versus PLA and INU in prediabetic individuals. The lack of increased breath H_2_ excretion after INU+RS intake suggests that microbial acetate production may have occurred earlier to the CID (e.g. in the more proximal colon) and microbially derived acetate remained for a prolonged period in the systemic circulation. In support of this theory, kinetic studies demonstrated that in metabolically disturbed individuals the clearance rate of intravenously infused acetate was lower and the half-life time was longer than in healthy individuals, indicating a disturbed uptake into peripheral tissues and/or catabolism of acetate.^22^

Of note, in accordance with our findings, several human studies indicate that in healthy individuals the metabolic response after oral SCFA administration, intravenous acetate, or vinegar interventions is more effective in improving glucose homeostasis and insulin sensitivity, than in metabolically compromised phenotypes (including metabolic syndrome, hyperinsulinemia and T2DM).^22–25,43^ This raises the questions whether the gut microbiota and thereby SCFA metabolism is altered in the microbiome of obese/prediabetic individuals and/or that SCFA signaling pathways are disrupted in the obese prediabetic phenotype (‘SCFA resistance’). Furthermore, can such potentially modified microbiota and SCFA production be reversed on the long term by dietary fiber treatment. Besides reduced acetate clearance, another reason for the increase in circulating acetate in prediabetic individuals, but not in butyrate, may relate to slower production of acetate in the colon and/or lowered conversion from acetate to butyrate due to cross-feeding mechanism in the (distal) colon.^44,45^

The metabolic effect with INU+RS in lean individuals seems to be fiber-specific, as these effects were not observed with BG+RS, which may relate to a greater substrate-specific shift in microbial composition with INU+RS as compared to BG+RS. Only a few colonic microbes are able to use long-chain or complex carbohydrates, with many more microbes benefiting from release of short-chain intermediary products by these primary degraders. For instance, only a few bifidobacterial species have the capacity to utilize long-chain insulin.^46,47^ Of interest, when we analyzed ASVs belonging to the *Bifidobacterium* genus, on average (although not significant) the abundance of ASVs tended to be higher in the microbiota of the lean versus prediabetic individuals (supplementary figure 6). Future studies should investigate whether a more prolonged consumption of the INU+RS fiber mixture increases the capacity of the distal colonic microbiota to metabolize more complex fibers to SCFA and improve metabolic health in both lean as well as overweight/obese prediabetic individuals.

In contrast to our in vitro findings and our hypothesis, we were not able to detect an increase in fecal or plasma acetate concentrations after INU+RS intake in the lean group. A plausible reason may be the differences in in vitro (effect of fiber mixtures on SCFA production per se) and in vivo experimental conditions (effect of fiber mixture on SCFA and metabolism the day after). In vivo, splanchnic extraction but also a rapid uptake of acetate in peripheral tissues such as skeletal muscle and adipose tissue might be one of the explanations why no increase in circulating acetate concentrations was detectable in lean participants. Another reason may be that a substantial amount of microbial produced acetate was converted via microbial cross feeding into other SCFA or additional metabolites, as shown via stable isotope techniques in rodents and humans.^48,49^ Of note, we found a slight increase in fasting plasma butyrate concentrations after INU+RS in the lean phenotype. This is remarkable, since as demonstrated in a kinetic study,^33^ only a very small proportion (~2%) of butyrate reaches the circulation when produced in the proximal colon, whereas the majority is directly used by colonocytes as energy source or metabolized in the liver. Thus, the increase in circulating butyrate together with the increased breath H_2_ suggest that the addition of the RS resulted in delayed fermentation of the long-chain inulin, which may have (partly) occurred in the distal colon. Indeed, SCFA produced in the distal part can partly bypass the liver via rectal veins and can therefore reach the systemic circulation directly in higher concentrations.^5,50^ In addition, the SCFA release from the distal colon (leading to increased SCFA concentrations in the systemic circulation) is higher compared to the proximal colon.^51^ Importantly, fasting and postprandial PYY concentrations were only increased after INU+RS intake in lean men. The increased breath H_2_ and circulating butyrate concentrations coincide with increased plasma PYY concentrations. This outcome is another indication that distal colonic saccharolytic fermentation occurred, since previous acute human studies showed that after distal,^17,18,52^ but not proximal,^17^ colonic infusions of acetate and SCFA mixtures, circulating PYY levels were increased. This can be explained by both a higher density of PYY secreting enteroendocrine cells as well as an increased expression of GPR43 in the distal colon as compared to the proximal colon.^53–55^

These well-controlled crossover studies have limitations. SCFA production by a complex mixture of microbes is the net effect of several processes, i.e. production and uptake by the microbiota itself and uptake by the host. Therefore, increased circulating SCFA might be the result of increased microbial fermentation or the effect of endogenous processes contributing to SCFA fluxes. In future experiments, isotopically labeled fibers might be used to answer this research question. In addition, the subacute design of these studies using only male participants can be seen as a limitation as it remains to be determined whether the metabolic outcomes translate into long-term relevance and can be translated to a female population. Since the study was powered on the predetermined primary outcome plasma acetate concentrations, which was non-significant in the INU for lean and BG for both phenotype, we cannot exclude that this can impact the interpretation of the secondary outcome (e.g. glucose homeostasis and substrate oxidation) in both clinical trials. In the INU study, we found important differences in metabolic outcomes between the subgroups but cannot be sure that this is related to distal colonic acetate production as hypothesized. The metabolic outcomes may therefore relate to other mechanisms such as an increased conversion in the lean group to butyrate.

In conclusion, our data demonstrated that a one-day supplementation of a fiber mixture of a ‘rapidly fermentable’ RS combined with a ‘slowly fermentable’ INU versus INU and PLA increased distal colonic bacterial saccharolytic fermentation the morning after the supplementation day in lean individuals. This enhanced bacterial fermentation coincides with an increased postprandial energy expenditure and carbohydrate oxidation, improved postprandial insulin sensitivity and increased circulating PYY concentrations. However, in overweight/obese prediabetic individuals INU+RS increased circulating acetate concentrations versus INU and PLA but did not result in improvements in the metabolic health parameters. These acute metabolic effects appeared to be fiber specific since BG+RS did not affect fermentation markers or metabolic outcomes in lean men when compared to BG alone or placebo. Whereas the in vitro TIM-2 study showed a direct shift in microbial SCFA production from BG+RS, the intervention was possibly too short to induce sustained metabolic effects in the study participants with this fiber combination. Fecal microbiota was affected by both treatments, but the shift in composition was highly individual and tended to be fiber-specific with more pronounced effects with INU+RS as compared to BG+RS. These results suggest that using INU+RS might be a nutritional tool to prevent body weight gain and insulin resistance, given that our findings are translatable to long-term conditions. Additionally, our data also open the gate for more individualized and subgroup-specific fiber interventions. Further research has to elucidate whether in the prediabetic phenotype, a longer-term supplementation period is required to demonstrate beneficial effects on metabolic health.

## Supplementary Material

Supplemental Material

## Data Availability

Sequencing data will be deposited in the European Nucleotide Archive (PRJEB47404).

## Abbreviations

ASVs: Amplicon sequence variants; BG: beta glucan; BMI: body mass index; CID: Clinical investigation day; FFA: Free fatty acid; GOS: Galacto-oligosaccharides; GLP-1: Glucagon-like peptide-1; GPR: G-protein coupled receptor; IFG: Impaired fasting glucose; IGT: Impaired glucose tolerance; INU: long-chain inulin; OGTT: Oral glucose tolerance test; PYY: Peptide YY; RS: Resistant starch; RQ, Respiratory quotient; SCFA: Short-chain fatty acid; T2DM: Type 2 diabetes.
