# Supplementary material for: Fiber mixture-specific effect on distal colonic fermentation and metabolic health in lean but not in prediabetic men
Source: Gut Microbes. 2021 Dec 20;14(1):2009297. doi: 10.1080/19490976.2021.2009297 (PMC8726743; doi:10.1080/19490976.2021.2009297)
Supplement: Supplemental Material [file KGMI_A_2009297_SM6831.zip › supplementary/Supplementary_figures_and_tables_CLEAN.docx]

**Supplementary figures**

**Supplementary figure 1**. Co-ingestion of inulin and resistant starch increased acetate and butyrate production with lean, but not with prediabetic microbiota. The TIM-2 system was either incubated with microbiota from lean (A, C, E, G) or prediabetic donors (B, D, F, H). Dashed lines (and the values after the +-symbol) indicate (quantitative) changes of SCFA production in the last 16 hours of incubation reflecting the distal colon. RS, resistant starch; SCFA, short-chain fatty acids

**Supplementary figure 2**. Co-ingestion of GOS and resistant starch with lean and prediabetic microbiota. The TIM-2 system was either incubated with microbiota from lean (A,C,E,G) or prediabetic donors (B,D,F,H). Dashed lines (and the values after the +-symbol) indicate (quantitative) changes of SCFA production in the last 16 hours of incubation reflecting the distal colon. RS, resistant starch; SCFA, short-chain fatty acids

**Supplementary figure 3**. Co-ingestion of beta glucan and resistant starch increased acetate production with lean, but not with prediabetic microbiota. The TIM-2 system was either incubated with microbiota from lean (A,C,E,G) or prediabetic donors (B,D,F,H). Dashed lines (and the values after the +-symbol) indicate (quantitative) changes of SCFA production in the last 16 hours of incubation reflecting the distal colon. RS, resistant starch; SCFA, short-chain fatty acids

**Supplementary figure 4. Fecal SCFA concentrations after fiber intake.** Fecal concentrations of total SCFA of lean (A) and prediabetic (B), acetate of lean (C) and prediabetic (D), propionate of lean (E) and prediabetic (F) and butyrate of lean (G) and prediabetic (H) individuals of the inulin study. Fecal concentrations of total SCFA of lean (I) and prediabetic (J), acetate of lean (K) and prediabetic (L), propionate of lean (M) and prediabetic (N) and butyrate of lean (O) and prediabetic (P) individuals of the beta glucan study. For the statistical analysis fecal samples of 8 lean (four individuals were not able to sample feces on all 3 days) and 10 prediabetic (one individual was not able to sample feces on all day) men in the inulin study and 10 lean (one individual was not able to sample feces on all day) and 11 prediabetic men in the beta glucan study was used. RS, resistant starch; BG, beta glucan; INU, long-chain inulin; PLA, placebo.

**Supplementary figure 5. Bargraphs of the top 12 most abundant bacterial families of each individual after supplementation of INU, INU+RS and PLA (A+B) as well as BG, BG+RS and PLA (C+D) in lean (A (n=8, four individuals were not able to sample feces on all 3 days), C (n=10, one individual was not able to sample feces on all days) and prediabetic men (B (n=10, one individual was not able to sample feces on all 3 days), D (n=11)).** RS, resistant starch; BG, beta glucan; INU, long-chain inulin; PLA, placebo

**
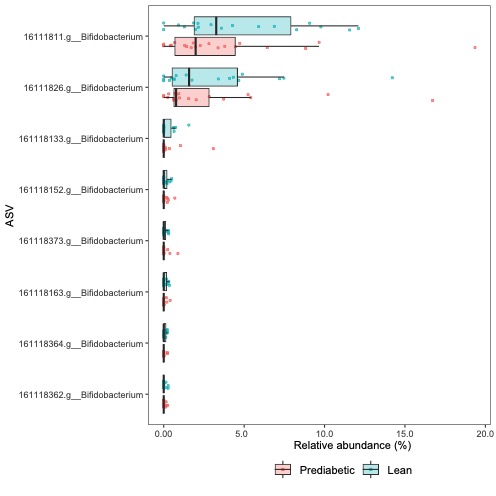
**

**Supplementary figure 6. Boxplots of the relative abundance of Bifidobacterium ASVs after PLA intervention in lean (n=8, four individuals were not able to sample feces on all 3 days) and prediabetic (n=10, one individual was not able to sample feces on all 3 days), individuals participating in the inulin and beta glucan studies.** Shown are the most important ASVs with a relative abundance of >0.1% in at least 20% of participants.

**Supplementary tables**

**Food intake**

Three-day food diaries were obtained prior to each CID.

**Inulin study**

One participant of the lean group was excluded from dietary intake analysis due to incomplete documentation of food intake. Energy intake and macronutrient composition on the first two days and third day was not different between intervention groups in lean and prediabetic individuals (supplementary table 1A and 1B, respectively). As instructed, dietary fiber intake on day 3 (day of fiber administration) significantly increased with INU+RS and INU compared to PLA in lean and prediabetic participants (P < 0.001) and with INU+RS compared to INU (lean: P < 0.001; prediabetic: P = 0.003; Supplementary table 1A and 1B).

| Supplementary Table 1a: Three day food record. Energy intake and dietary composition in the inulin Study of lean Participants (n = 11) prior to CID. | | | | | |
| --- | --- | --- | --- | --- | --- |
| Variable | **Time** | **Treatment** | | | **P-value** |
|  |  | Placebo | Inulin | Inulin + RS |  |
| Energy intake (kcal) | Day 1+2 (mean) | 2381 ± 480 | 2287 ± 447 | 2333 ± 467 | 0.84 |
|  | Day 3 | 2312 ± 408 | 2434 ± 703 | 2524 ± 420 | 0.23 |
| Carbohydrate intake (g) | Day 1+2 (mean) | 280 ± 61 | 280 ± 67 | 274 ± 71 | 0.97 |
|  | Day 3 | 289 ± 54 | 308 ± 74 | 320 ± 74 | 0.17 |
| Total fat intake (g) | Day 1+2 (mean) | 88 ± 30 | 82 ± 22 | 82 ± 20 | 0.77 |
|  | Day 3 | 89 ± 21 | 90 ± 38 | 91 ± 17 | 0.73 |
| Saturated fat intake (g) | Day 1+2 (mean) | 34 ± 17 | 31 ± 11 | 31 ± 8 | 0.86 |
|  | Day 3 | 43 ± 9 | 41 ± 14 | 42 ± 10 | 0.78 |
| Protein intake (g) | Day 1+2 (mean) | 88 ± 24 | 92 ± 19 | 91 ± 20 | 0.90 |
|  | Day 3 | 78 ± 19 | 80 ± 27 | 80 ± 11 | 0.87 |
| Fiber intake (g) | Day 1+2 (mean) | 26 ± 10 | 28 ± 9 | 28 ± 15 | 0.88 |
|  | Day 3 | 17 ± 5^a^ | 30 ± 5^b^ | 40 ± 6^c^ | <0.001* |
| *Values are given as mean ± standard deviation. P-values were obtained by linear mixed model analysis. Significance was set at p < 0.05. Significant values are indicated by asterisk (*).* RS, resistant starch. | | | | | |

| Supplementary Table 1b: Three day food record. Energy intake and dietary composition in the Inulin of PREDIABETIC Participants (n = 11) prior to CID. | | | | | | |
| --- | --- | --- | --- | --- | --- | --- |
| Variable | **Time** | **Treatment** | | | ***P*-value** | |
|  |  | **Placebo** | **Inulin** | **Inulin + RS** |  | |
| Energy intake (kcal) | Day 1+2 (mean) | 2206 ± 538 | 2470 ± 805 | 2202 ± 412 | 0.36 |  |
|  | Day 3 | 2117 ± 526 | 2147 ± 454 | 2109 ± 431 | 0.86 |  |
| Carbohydrate intake (g) | Day 1+2 (mean) | 221 ± 42 | 274 ± 80 | 225 ± 33 | 0.04 |  |
|  | Day 3 | 275 ± 87 | 270 ± 67 | 267 ± 53 | 0.83 |  |
| Total fat intake (g) | Day 1+2 (mean) | 76 ± 28 | 84 ± 46 | 82 ± 22 | 0.77 |  |
|  | Day 3 | 75 ± 21 | 75 ± 23 | 73 ± 22 | 0.99 |  |
| Saturated fat intake (g) | Day 1+2 (mean) | 25 ± 10 | 29 ±17 | 27 ± 10 | 0.63 |  |
|  | Day 3 | 35 ± 10 | 35 ± 9 | 34 ± 10 | 0.98 |  |
| Protein intake (g) | Day 1+2 (mean) | 101 ± 33 | 92 ± 28 | 88 ± 26 | 0.34 |  |
|  | Day 3 | 73 ± 25 | 77 ± 21 | 74 ± 18 | 0.75 |  |
| Fiber intake (g) | Day 1+2 (mean) | 21 ± 7 | 24 ± 8 | 22 ± 7 | 0.36 |  |
|  | Day 3 | 17 ± 4^a^ | 30 ± 4^b^ | 37 ± 6^c^ | <0.001* |  |
| *Values are given as mean ± standard deviation. P-values were obtained by linear mixed model analysis. Significance was set at p < 0.05*. *Significant values are indicated by asterisk (*).* RS, resistant starch. | | | | | | |

**Beta glucan study**

Two participants of the lean group were excluded from dietary intake analysis due to incomplete documentation of food intake. Energy intake and macronutrient composition on the first two days and third day was not significantly different between intervention groups in lean nor in prediabetic individuals (supplementary table 1C and 1D, respectively). Dietary fiber intake on day 3 (day of fiber administration) significantly increased with BG and BG+RS compared to PLA (P < 0.001) and with BG+RS compared to BG (P < 0.001; Supplementary table 1C and 1D) in lean and prediabetic participants.

| Supplementary Table 1C: Three day food record. Energy intake and dietary composition in the beta glucan study of lean Participants (n = 9) prior to CID. | | | | | | |
| --- | --- | --- | --- | --- | --- | --- |
| Variable | **Time** | **Treatment** | | | ***P*-value** | |
|  |  | Placebo | BG | BG + RS |  | |
| Energy intake (kcal) | Day 1+2 (mean) | 2226 ± 431 | 2318 ± 449 | 2153 ± 400 | .91 |  |
|  | Day 3 | 2220 ± 453 | 2008 ± 503 | 2218 ± 276 | .25 |  |
| Carbohydrate intake (g) | Day 1+2 (mean) | 236 ± 39 | 248 ± 57 | 252 ± 24 | .30 |  |
|  | Day 3 | 238 ± 68 | 239 ± 66 | 230 ± 47 | .58 |  |
| Total fat intake (g) | Day 1+2 (mean) | 83 ± 24 | 103 ± 32 | 79 ± 28 | .32 |  |
|  | Day 3 | 79 ± 25 | 65 ± 32 | 80 ± 29 | **.**14 |  |
| Saturated fat intake (g) | Day 1+2 (mean) | 32 ± 11 | 36 ± 11 | 30 ± 13 | .69 |  |
|  | Day 3 | 30 ± 12 | 27 ± 18 | 32 ± 13 | .64 |  |
| Protein intake (g) | Day 1+2 (mean) | 92 ± 24 | 90 ±29 | 78 ± 22 | .65 |  |
|  | Day 3 | 109 ± 22 | 100 ± 26 | 109 ± 18 | .18 |  |
| Fiber intake (g) | Day 1+2 (mean) | 24± 8 | 26 ± 7 | 25 ± 6 | .93 |  |
|  | Day 3 | 15 ± 4 | 26 ± 4 | 35 ± 4 | **<.001*** |  |
| *Values are given as mean ± standard deviation. P-values were obtained by linear mixed model analysis. Significance was set at p < 0.05*. *Significant values are indicated by asterisk (*).* BG, beta glucan; RS, resistant starch. | | | | | | |

| Supplementary Table 1D: Three day food record. Energy intake and dietary composition in the beta glucan study of predaiabetic Participants (n = 11) prior to CID. | | | | | | |
| --- | --- | --- | --- | --- | --- | --- |
| Variable | **Time** | **Treatment** | | | ***P*-value** | |
|  |  | Placebo | BG | BG + RS |  | |
| Energy intake (kcal) | Day 1+2 (mean) | 2196 ± 632 | 2326 ± 599 | 2256 ± 510 | .76 |  |
|  | Day 3 | 1875 ± 473 | 2021 ± 568 | 2157± 560 | .18 |  |
| Carbohydrate intake (g) | Day 1+2 (mean) | 218 ± 72 | 223 ± 61 | 212 ± 37 | .40 |  |
|  | Day 3 | 197 ± 54 | 227 ± 70 | 218 ± 56 | .38 |  |
| Total fat intake (g) | Day 1+2 (mean) | 87 ± 31 | 99 ± 50 | 92 ± 33 | .42 |  |
|  | Day 3 | 64 ± 26 | 64 ± 26 | 76 ± 35 | .34 |  |
| Saturated fat intake (g) | Day 1+2 (mean) | 30 ± 10 | 37 ± 21 | 34 ± 12 | .36 |  |
|  | Day 3 | 23 ± 11 | 21 ± 9 | 28 ± 18 | .29 |  |
| Protein intake (g) | Day 1+2 (mean) | 104 ± 28 | 99 ± 27 | 107 ± 34 | .59 |  |
|  | Day 3 | 103 ± 17 | 109 ± 26 | 121 ± 21 | **.**12 |  |
| Fiber intake (g) | Day 1+2 (mean) | 21 ± 8 | 22 ± 6 | 21 ± 5 | .84 |  |
|  | Day 3 | 15 ± 1 | 28 ± 5 | **35 ± 5** | **<.001*** |  |
| *Values are given as mean ± standard deviation. P-values were obtained by linear mixed model analysis. Significance was set at p < 0.05*. *Significant values are indicated by asterisk (*).* BG, beta glucan; RS, resistant starch. | | | | | | |

| Supplementary table 2: Plasma SCFA concentrations and breath H_2_ excretion | | | | | | |  |  |  |  |  |  |  |  |  |  |  |
| --- | --- | --- | --- | --- | --- | --- | --- | --- | --- | --- | --- | --- | --- | --- | --- | --- | --- |
| *Inulin study* |  |  |  |  |  |  |  |  |  |  |  |  |  |  |  |  |  |
| Phenotype | **Variables** | **Fasted** |  |  |  | **total AUC** |  |  |  | **AUC 0-120** | |  |  | **AUC120-240** | |  |  |
|  |  | PLA | INU | INU+RS | P-value | PLA | INU | INU+RS | P-value | PLA | INU | INU+RS | P-value | PLA | INU | INU+RS | P-value |
| Lean | **Acetate, μmol/L** | 57.67  ± 1.56 | 72.07  ± 2.29 | 57.14  ± 1.79 | 0.141 | 11256.92  ± 305 | 11730  ± 197 | 11034  ± 319 | 0.69 | 5576.22 ±128 | 6140.48  ± 126 | 5405.13  ± 168 | 0.263 | 5680.71  ± 187 | 5590.31  ± 95 | 5629.25  ± 163 | 0.895 |
|  | **Propionate, μmol/L** | 1.01  ± 0.07 | 1.21  ± 0.09 | 1.21  ± 0.08 | 0.471 | 359.54  ± 28.7 | 317.52  ± 24.7 | 314.61  ± 21.5 | 0.474 | 183.04  ± 15 | 164.21  ± 13 | 172.74  ± 13 | 0.57 | 176.50  ± 14 | 153.30  ± 12 | 141.86  ± 9 | 0.363 |
|  | **Butyrate, μmol/L** | 0.43  ± 0.02 | 0.57  ± 0.02 | **0.68**  **± 0.03*#** | **0.044** | 215.11  ± 7.4 | 201.33  ±4.4 | 198.30  ± 8.4 | 0.931 | 132.45  ± 4 | 124.47  ± 4 | 132.0  ± 6 | 0.784 | 131.69  ± 5 | 124.81  ± 3 | 120.17  ± 5 | 0.914 |
|  | **Breath H2, ppm** | 15.77  ± 1.32 | 22.08  ± 1.09 | **32.33**  **± 2.58*** | **0.052** | 2629.25  ± 168 | 3746.25  ± 152 | **4257.50**  **± 220*** | **0.045** | 1542.96 ± 108 | 2213.75  ± 97 | **2813.75**  **± 71*** | **0.028** | 1086.3  ± 65 | 1532.5  ± 68 | 1443.75  ± 71 | 0.128 |
| Prediabetic | **Acetate, μmol/L** | 54.42  ± 2.14 | 54.87  ± 2.65 | **68.82 ± 2.06*** | **0.061** | 9482.11  ± 350 | 10545.04  ± 464 | **12379.17**  **± 405*** | **0.019** | 5157.32 ± 190 | 5519.49  ± 269 | **6414.21**  **± 216*** | **0.041** | 4324.79  ± 165 | 5025.56  ± 200 | **5964.96**  **± 216*** | **0.015** |
|  | **Propionate, μmol/L** | 1.06  ± 0.04 | 0.94  ± 0.05 | 1.04 ±  0.04 | 0.831 | 256.3  ± 7.7 | 266.75  ± 8.2 | 280.42  ± 10.6 | 0.831 | 143.47  ± 4 | 141.92  ± 6 | 147.76  ± 5 | 0.998 | 112.79  ± 4 | 124.83  ± 3 | 132.66  ± 7 | 0.733 |
|  | **Butyrate, μmol/L** | 0.53  ± 0.04 | 0.042  ± 0.03 | 0.063 ± 0.06 | 0.377 | 207.45  ± 12.7 | 220.5  ± 12.2 | 240.5  ± 20.1 | 0.603 | 115.77  ± 8 | 106.75  ± 6 | 128.86  ± 10 | 0.321 | 91.68  ± 5 | 113.11  ± 7 | 111.63  ± 11 | 0.428 |
|  | **Breath H2, ppm** | 21.09  ± 1.17 | 35.14  ± 3.85 | 19.18 ± 1.21 | 0.108 | 3725.46  ± 205 | 5697.95  ± 570 | 3287.73  ± 194 | 0.386 | 2214.54  ± 106 | 3409.77  ± 353 | 1309.63 ±114 | 0.176 | 1510.91 ±117 | 2288.18  ± 251 | 1309.09  ± 83 | 0.119 |
| *Beta glucan study* | |  |  |  |  |  |  |  |  |  |  |  |  |  |  |  |  |
|  |  | PLA | BG | BG+RS |  | PLA | BG | BG+RS |  | PLA | BG | BG+RS |  | PLA | BG | INU+RS |  |
| Lean | **Acetate, μmol/L** | 67.63  ± 3.22 | 64.8  ± 3.03 | 68.64  ± 2.88 | 0.672 | 10253.34  ± 264 | 10936.34  ± 385 | 11524.45  ± 407 | 0.164 | 5124.07  ± 174 | 5517.72  ± 215 | 5798  ± 240 | 0.153 | 5129.27  ± 99 | 5418.62  ± 215 | 5726.7  ± 179 | 0.434 |
|  | **Propionate, μmol/L** | 1.52  ± 0.1 | 1.77  ± 0.11 | 1.8  ± 0.1 | 0.788 | 388.73  ± 15 | 495  ± 26 | 515.63  ± 20 | 0.147 | 206.24  ± 9 | 250.36  ± 12 | 268  ± 14 | 0.147 | 182.49  ± 7 | 244.63  ± 15 | 247.09  ± 7 | 0.255 |
|  | **Butyrate, μmol/L** | 0.55  ± 0.04 | 0.67  ± 0.05 | 0.76  ± 0.05 | 0.321 | 296.76  ± 12 | 323  ± 14 | 357.76  ± 12 | 0.165 | 159.96  ± 8 | 156.22  ± 7 | 187.42  ± 10 | 0.253 | 136.8  ± 4 | 166.75  ± 8 | 170.36  ± 4 | 0.180 |
|  | **Breath H2, ppm** | 17.36  ± 1.26 | 10.36  ± 0.65 | 12.14  ± 0.69 | 0.165 | 3301.36  ± 223 | 1981.36  ± 77 | 2035.23  ± 74 | 0.099 | 2035.91  ± 164 | 1204.09  ± 63 | 1249.77  ± 58 | 0.2 | 1265.46  ± 67 | **777.27**  **± 23*** | **785.46**  **± 21*** | **0.020** |
| Prediabetic | **Acetate, μmol/L** | 51.46  ± 3.19 | 57.96  ± 4.29 | 51.19  ± 3.24 | 0.181 | 9545.5  ± 548 | 88817.66  ± 469 | 8004.24  ± 452 | 0.350 | 5215.65  ± 283 | 4968.27  ± 268 | 4492.98  ± 231 | 0.475 | 4329.85  ± 274 | 3849.38  ± 207 | 3511.26  ± 223 | 0.311 |
|  | **Propionate, μmol/L** | 0.87  ± 0.07 | 1.14  ± 0.07 | 0.97  ± 0.06 | 0.363 | 247.7  ± 13 | 323.16  ± 11 | 269.28  ± 8 | 0.113 | 130.6  ± 7 | 176.44  ± 7 | 161.5  ± 6 | 0.058 | 117.08  ± 7 | 146.71  ± 5 | 107.78  ± 3 | 0.136 |
|  | **Butyrate, μmol/L** | 0.51  ± 0.03 | 0.64  ± 0.05 | 0.78  ± 0.07 | 0.107 | 217.07  ± 10 | 281.84  ± 14 | **331.5**  **± 20*** | **0.037** | 114.88  ± 5 | 150.55  ± 8 | **173.13**  **±12*** | **0.031** | 102.2  ± 5 | 131.29  ± 6 | 140.36  ± 9 | 0.096 |
|  | **Breath H2, ppm** | 10.09  ± 067 | 9.32  ± 0.03 | 10.05  ± 0.03 | 0.826 | 1808.18  ± 66 | 1919.32  ± 8 | 1991.59  ± 8 | 0.984 | 1107.27  ± 47 | 1120.23  ± 9 | 1214.32  ± 9 | 0.931 | 700.91  ± 24 | 799.09  ±11 | 777.27  ± 11 | 0.966 |
| Values are presented as mean ± Standard error of the mean (S.E.M.) * P<0.05 versus placebo, # P<0.05 versus inulin alone. Differences in fasting (t0) and postprandial AUC between the three interventions (PLA, INU, and INU+RS or PLA, BG and BG+RS) were analyzed using a linear mixed model for repeated measures. | | | | | | | | | | | | | | | | |  |

| Supplementary table 3: Energy expenditure and substrate oxidation | | | | | | |  |  |  |  |  |  |  |  |  |  |  |
| --- | --- | --- | --- | --- | --- | --- | --- | --- | --- | --- | --- | --- | --- | --- | --- | --- | --- |
| *Inulin study* | | |  |  |  |  |  |  |  |  |  |  |  |  |  |  |  |
| Phenotype | **Variables** | **Fasted** |  |  |  | **total AUC** |  |  |  | **AUC 0-120** | |  |  | **AUC120-240** | |  |  |
|  |  | PLA | INU | INU+RS | P-value | PLA | INU | INU+RS | P-value | PLA | INU | INU+RS | P-value | PLA | INU | INU+RS | P-value |
| Lean | **EE, kJ/min** | 4.66  ± 0.12 | 4.72  ± 0.14 | 4.82  ± 016 | 0.296 | 1250  ± 29 | 1272  ±31 | **1299**  **± 39*** | **0.062** | 630  ± 17 | 636  ± 17 | 649  ± 20 | 0.157 | 463  ± 9 | 478  ± 10 | **490**  **± 14**** | **0.017** |
|  | **Fat oxidation,**  **g/min** | 0.060  ± 0.005 | 0.058  ± 0.006 | **0.045**  **± 0.006*** | **0.064** | 12.69  ± 0.97 | 12.96  ± 1.23 | 10.31  ± 1.06 | 0.142 | 5.98  ± 0.53 | 5.89  ± 0.55 | 4.59  ± 0.5 | 0.116 | 5.12  ± 0.54 | 5.58  ± 0.60 | 4.45  ± 0.41 | 0.180 |
|  | **Carbohydrate oxidation, g/min** | 0.095  ± 0.017 | 0.102  ± 0.021 | **0.140**  **± 0.021*** | **0.060** | 34.05  ± 3.08 | 34.23  ± 3.28 | **42.60**  **± 3.73*** | **0.070** | 18.21  ± 1.84 | 19.01  ±1.78 | **22.77**  **± 1.87*** | **0.074** | 11.58  ± 1.04 | 10.90  ± 1.34 | 14.55  ± 1.41 | 0.078 |
|  | **RQ** | 0.811  ± 0.015 | 0.0812  ± 0.018 | **0.850**  **± 0.017*** | **0.048** | 202.95  ± 2 | 201.93  ± 3 | 208.34  ± 2 | 0.092 | 101.79  ± 1 | 102.37  ± 1 | 105.3  ± 1 | 0.085 | 75.83  ± 1 | 75.05  ± 1 | 77.77  ± 1 | 0.120 |
| Prediabetic | **EE, kJ/min** | 5.33  ± 0.18 | 5.29  ± 0.20 | 5.25  ± 0.15 | 0.796 | 1422  ± 43 | 1423  ± 44 | 1400  ± 41 | 0.617 | 714  ± 24 | 714  ± 24 | 707  ± 22 | 0.949 | 530  ± 14 | 532  ± 15 | 522  ± 15 | 0.487 |
|  | **Fat oxidation,**  **g/min** | 0.079  ± 0.004 | 0.076  ± 0.005 | 0.074  ± 0.003 | 0.774 | 16.06  ± 0.78 | 15.36  ± 1.07 | 16.26  ± 0.89 | 0.182 | 7.88  ± 0.44 | 7.39  ± 0.58 | 7.70  ± 0.50 | 0.358 | 6.28  ± 0.43 | 6.15  ± 0.42 | 6.60  ± 0.36 | 0.179 |
|  | **Carbohydrate oxidation, g/min** | 0.083  ± 0.012 | 0.088  ± 0.010 | 0.094  ± 0.011 | 0.852 | 34.52  ± 2.19 | 36.32  ± 2.27 | 32.96  ± 2.59 | 0.177 | 17.83  ± 1.48 | 19.07  ± 1.29 | 18.04  ± 1.51 | 0.554 | 12.13  ± 1.01 | 12.44  ± 1.00 | 10.089  ± 1.02 | 0.169 |
|  | **RQ** | 0.790  ± 0.009 | 0.798  ± 0.010 | 0.800  ± 0.008 | 0.756 | 199.21  ± 1 | 200.68  ± 2 | 198.38  ± 2 | 0.190 | 99.92  ± 1 | 101.03  ± 1 | 100.21  ± 1 | 0.392 | 75.02  ± 1 | 75.17  ± 1 | 74.19  ± 1 | 0.133 |
| *Beta glucan study* | |  |  |  |  |  |  |  |  |  |  |  |  |  |  |  |  |
|  |  | PLA | BG | BG+RS |  | PLA | BG | BG+RS |  | PLA | BG | BG+RS |  | PLA | BG | INU+RS |  |
| Lean | **EE, kJ/min** | 4.80  ± 0.15 | 4.85  ± 0.15 | 4.95  ± 0.15 | 0.175 | 1291  ± 38 | 1316  ± 43 | 1318  ± 40 | 0.546 | 653  ± 20 | 659  ± 24 | 661  ± 23 | 0.903 | 638  ± 18 | 657  ± 20 | 657  ± 17 | 0.308 |
|  | **Fat oxidation,**  **g/min** | 0.062  ± 0.005 | 0.063  ± 0.006 | 0.053  ± 0.006 | 0.168 | 12.90  ± 0.92 | 11.67  ± 0.95 | 11.15  ± 1.10 | 0.194 | 6.02  ± 0.51 | 5.55  ± 0.48 | 5.33  ± 0.56 | 0.318 | 6.87  ± 0.45 | 6.12  ± 0.48 | 5.83  ± 0.56 | 0.173 |
|  | **Carbohydrate oxidation, g/min** | 0.097  ± 0.013 | 0.101  ± 0.020 | 0.128  ± 0.017 | 0.132 | 35.91  ± 2.75 | 40.27  ±3.67 | 41.57  ± 3.26 | 0.166 | 19.32  ± 1.48 | 20.91  ± 1.92 | 21.55  ± 1.66 | 0.326 | 16.60  ±1.40 | 19.31  ± 1.87 | 20.02  ± 1.76 | 0.117 |
|  | **RQ** | 0.812  ± 0.012 | 0.811  ± 0.017 | 0.837  ± 0.016 | 0.100 | 202.72  ± 2 | 205.58  ± 3 | 207.04  ± 2 | 0.113 | 102.36  ± 1 | 103.35  ± 1 | 104.08  ± 1 | 0.203 | 100.36  ± 1 | 102.223  ± 1 | 102.97  ± 1 | 0.092 |
| Prediabetic | **EE, kJ/min** | 5.42  ± 0.17 | 5.33  ±0.19 | 5.44  ± 0.20 | 0.468 | 1418  ± 43 | 1423  ± 40 | 1433  ± 47 | 0.386 | 713  ± 20 | 710  ± 19 | 720  ± 24 | 0.967 | 705  ± 23 | 713  ± 23 | 724  ± 24 | 0.123 |
|  | **Fat oxidation,**  **g/min** | 0.076  ± 0.005 | 0.074  ± 0.006 | 0.070  ± 0.006 | 0.740 | 16.02  ± 1.06 | 15.54  ± 1.20 | 14.70  ± 1.08 | 0.600 | 7.98  ± 0.57 | 7.41  ± 0.68 | 7.05  ± 0.68 | 0.344 | 8.04  ± 0.52 | 8.14  ± 0.58 | 7.65  ± 0.47 | 0.762 |
|  | **Carbohydrate oxidation, g/min** | 0.094  ± 0.013 | 0.093  ± 0.013 | 0.112  ± 0.014 | 0.603 | 34.38  ± 2.54 | 35.36  ± 3.09 | 38.36  ± 2.54 | 0.298 | 17.52  ± 1.47 | 18.77  ± 1.88 | 19.74  ± 1.75 | 0.347 | 16.86  ± 1.14 | 16.59  ± 1.33 | 18.62  ± 0.99 | 0.218 |
|  | **RQ** | 0.799  ± 0.010 | 0.804  ± 0.014 | 0.814  ± 0.011 | 0.809 | 198.63  ± 2 | 199.07  ± 2 | 201.53  ± 2 | 0.414 | 99.59  ± 1 | 101.08  ± 2 | 101.68  ± 1 | 0.538 | 99.04  ± 1 | 97.99  ± 1 | 99.85  ± 1 | 0.215 |
| Values are presented as mean ± S.E.M. * P<0.05 versus placebo, ** P<0.01 versus placebo. EE, energy expenditure; RQ respiratory quotient. Differences in fasting (t0) and postprandial AUC between the three interventions (PLA, INU, and INU+RS or PLA, BG and BG+RS) were analyzed using a linear mixed model for repeated measures. | | | | | | | | | | | | | | | | |  |

| Supplementary table 4: Plasma metabolites and hormones | | | | | | |  |  |  |  |  |  |  |  |  |  |  |
| --- | --- | --- | --- | --- | --- | --- | --- | --- | --- | --- | --- | --- | --- | --- | --- | --- | --- |
| *Inulin study* |  |  |  |  |  |  |  |  |  |  |  |  |  |  |  |  |  |
| Phenotype | **Variables** | **Fasted** |  |  |  | **total AUC** |  |  |  | **AUC 0-120** | |  |  | **AUC120-240** | |  |  |
|  |  | PLA | INU | INU+RS | P-value | PLA | INU | INU+RS | P-value | PLA | INU | INU+RS | P-value | PLA | INU | INU+RS | P-value |
| Lean | **Glucose, mmol/L** | 5.25  ± 0.10 | 5.24  ± 0.06 | 5.12  ± 0.04 | 0.399 | 1296  ± 34 | 1281  ± 36 | 1230  ± 30 | 0.082 | 669  ± 20 | 664  ± 24 | **629**  **± 17*#** | **0.037** | 627  ± 15 | 616  ± 13 | 602  ± 14 | 0.212 |
|  | **FFA,**  **μmol/L** | 358  ± 68 | 361  ± 39 | 300  ± 38 | 0.182 | 62802  ± 6960 | 63543  ± 4755 | 58659  ± 4757 | 0.566 | 27065  ± 4436 | 27912  ± 2140 | 25027  ± 2941 | 0.596 | 35738  ± 3111 | 35631  ± 2988 | 33631  ± 2522 | 0.685 |
|  | **Insulin,**  **mU/L** | 6.44  ± 0.65 | 6.13  ± 0.60 | 6.43  ± 0.69 | 0.773 | 4029  ± 438 | 4033  ± 429 | 3968  ± 371 | 0.945 | 2649  ± 282 | 2691  ± 268 | 2666  ± 271 | 0.962 | 1380  ± 192 | 1342  ± 187 | 1302  ± 163 | 0.956 |
|  | **PYY,**  **pg/mL** | 14.83  ± 1.47 | 13.50  ± 1.41 | **17.62**  **± 2.05#** | **0.034** | 4543  ± 448 | 4673  ± 329 | **5703**  **± 483*#** | **0.029** | 2200  ± 206 | 2468  ± 235 | **2790**  **± 280*** | **0.052** | 2343  ± 279 | **2205**  **± 135** | **2913**  **± 223*#** | **0.026** |
|  | **GLP-1,**  **pmol/L** | 48.58  ± 3.23 | 46.17  ±2.40 | 49.25  ± 3.03 | 0.363 | 14169  ± 812 | 13876  ± 810 | 14433  ± 860 | 0.666 | 7471  ± 521 | 7021  ± 320 | 7355  ± 476 | 0.339 | 6698  ± 316 | 6855  ± 524 | 7078  ± 407 | 0.637 |
| Prediabetic | **Glucose, mmol/L** | 6.32  ± 0.23 | 6.28  ± 0.23 | 6.28  ± 0.26 | 0.953 | 1615  ± 42 | 1603  ± 42 | 1595  ± 49 | 0.907 | 879  ± 31 | 874  ± 32 | 883  ± 33 | 0.812 | 736  ± 17 | 730  ± 14 | 712  ± 21 | 0.514 |
|  | **FFA,**  **μmol/L** | 498  ± 29 | 499  ± 42 | 491  ± 34 | 0.967 | 76513  ± 4581 | 74240  ± 4628 | 77553  ± 4167 | 0.555 | 37547  ± 2755 | 37113  ± 2377 | 38093  ± 3010 | 0.834 | 38966  ± 3343 | 37126  ± 2967 | 39460  ± 2359 | 0.451 |
|  | **Insulin,**  **mU/L** | 13.85  ± 2.92 | 13.93  ± 2.60 | 13.19  ± 2.65 | 0.499 | 9794  ± 2014 | 9981  ± 1801 | 10151  ± 1934 | 0.938 | 6401  ± 1303 | 6320  ± 1026 | 6911  ± 1318 | 0.758 | 3393  ± 762 | 3661  ± 790 | 3240  ± 638 | 0.342 |
|  | **PYY,**  **pg/mL** | 13.27  ± 2.64 | 14.27  ± 1.06 | 16.09  ± 2.44 | 0.760 | 4944  ± 360 | 4426  ± 375 | 5493  ± 740 | 0.296 | 2481  ± 197 | 2223  ± 144 | 2779  ± 377 | 0.383 | 2463  ± 198 | 2204  ± 264 | 2714  ± 370 | 0.312 |
|  | **GLP-1,**  **pmol/L** | 48.00  ± 0.68 | 48.18  ± 0.59 | 47.73  ± 0.57 | 0.928 | 13930  ± 163 | 14775  ± 174 | 15177  ± 279 | 0.180 | 7392  ± 80 | 7801  ±101 | 7964  ± 181 | 0.375 | 6537  ± 90 | 6974  ± 82 | **7214**  **± 106*** | **0.083** |
| *Beta glucan study* | |  |  |  |  |  |  |  |  |  |  |  |  |  |  |  |  |
|  |  | PLA | BG | BG+RS |  | PLA | BG | BG+RS |  | PLA | BG | BG+RS |  | PLA | BG | INU+RS |  |
| Lean | **Glucose, mmol/L** | 5.17  ± 0.10 | 5.25  ± 0.11 | 5.14  ± 0.08 | 0.261 | 1242  ± 33 | 1232  ± 31 | 1220  ± 29 | 0.727 | 643  ± 24 | 643  ± 22 | 625  ± 20 | 0.709 | 599  ± 11 | 599  ± 13 | 596  ± 12 | 0.749 |
|  | **FFA,**  **μmol/L** | 370  ± 33 | 380  ± 33 | 330  ± 33 | 0.336 | 64985  ± 2958 | 61139  ± 3435 | 60563  ± 2543 | 0.445 | 27506  ± 2226 | 25915  ± 1713 | 24020  ± 1274 | 0.239 | 37479  ± 1611 | 35223  ± 2137 | 36543  ± 1800 | 0.601 |
|  | **Insulin,**  **mU/L** | 5.40  ± 0.37 | 6.04  ± 0.63 | 5.25  ± 0.37 | 0.251 | 3661  ± 415 | 3664  ± 395 | 3716  ± 383 | 0.498 | 2673  ± 306 | 2720  ± 290 | 2673  ± 313 | 0.678 | 988  ± 128 | 944  ± 121 | 1043  ± 121 | 0.464 |
|  | **GLP-1,**  **pmol/L** | 14.46  ± 0.65 | 14.73  ± 1.23 | 14.18  ± 0.78 | 0.959 | 4965  ± 270 | 4901  ± 222 | 4831  ± 206 | 0.796 | 2680  ± 165 | 2607  ± 136 | 2541  ± 111 | 0.462 | 2286  ± 148 | 2294  ± 100 | 2291  ± 122 | 0.902 |
| Prediabetic | **Glucose, mmol/L** | 6.11  ± 0.23 | 6.23  ± 0.17 | 6.23  ± 0.19 | 0.514 | 1533  ± 38 | 1498  ± 39 | 1510  ± 43 | 0.911 | 831  ± 31 | 820  ± 27 | 838  ± 33 | 0.589 | 703  ± 12 | 678  ± 19 | 673  ± 16 | 0.199 |
|  | **FFA,**  **μmol/L** | 531  ± 34 | 536  ± 55 | 465  ± 26 | 0.156 | 78353  ± 4448 | 84323  ± 5096 | 73962  ± 5154 | 0.086 | 39272  ± 2418 | 39294  ± 3430 | 35103  ± 2580 | 0.144 | 39080  ± 3026 | 45029  ± 2913 | **38859**  **± 3317$** | **0.074** |
|  | **Insulin,**  **mU/L** | 11.34  ± 1.68 | 13.15  ± 2.16 | 12.34  ± 1.82 | 0.126 | 7603  ± 1055 | 8064  ± 1540 | 8234  ± 1149 | 0.499 | 4836  ± 654 | 5417  ± 1048 | 5636  ± 858 | 0.169 | 2767  ± 429 | 2647  ± 508 | 2597  ± 321 | 0.686 |
|  | **GLP-1,**  **pmol/L** | 14.64  ± 1.80 | 14.73  ± 1.09 | 14.64  ± 0.85 | 0.914 | 5110  ± 239 | 5250  ± 318 | 5344  ± 386 | 0.662 | 2677  ± 111 | 2763  ± 160 | 2756  ± 191 | 0.686 | 2433  ± 147 | 2487  ± 166 | 2588  ± 212 | 0.671 |
| Values are presented as mean ± S.E.M. * P<0.05 versus placebo, # P<0.05 versus inulin alone, $ P<0.05 versus beta glucan alone. Differences in fasting (t0) and postprandial AUC between the three interventions (PLA, INU, and INU+RS or PLA, BG and BG+RS) were analyzed using a linear mixed model for repeated measures. | | | | | | | | | | | | | | | | |  |
